# Emotional Processing in Incarcerated Men and Women: Associations with Psychopathic Traits

**DOI:** 10.3390/bs16071160

**Published:** 2026-07-10

**Authors:** Daniela Ramos, Inês Lomba, Marina Leonor Pinheiro, Sónia Caridade, Marta Sousa, Olga Cunha

**Affiliations:** 1 Psychology Research Center (CIPsi), University of Minho, Campus de Gualtar, 4710-057 Braga, Portugal; daniela.sofia.ramos.oliveira@gmail.com (D.R.); ineslombaabc@gmail.com (I.L.); id10240@alunos.uminho.pt (M.L.P.); scaridade@psi.uminho.pt (S.C.); 2 HEI-Lab: Digital Human-Environment Interaction Lab, Lusófona University, Rua de Augusto Rosa 24, 4000-098 Porto, Portugal; martaasousaa@gmail.com

**Keywords:** emotional processing, psychopathic propensity, incarcerated men, incarcerated women, psychopathic traits

## Abstract

Psychopathy is characterized by callousness, manipulativeness and antisocial behavior and has been linked to impairments in emotional processing. Prior research suggests that psychopathic traits may manifest differently in men and women. However, few studies have compared incarcerated men and women on psychopathy and emotional processing. The present study examined the association between psychopathic traits and emotional processing in incarcerated men and women, with a particular focus on potential sex differences. The sample consisted of 144 male and 113 female individuals in Portuguese prisons. Data were collected using the Self-Report Psychopathy-Short Form (SRP-SF) and the Emotional Processing Scale-25 (EPS-25). Results indicated that men reported higher levels of psychopathic traits, particularly in the lifestyle and antisocial dimensions. Women reported greater difficulties in specific aspects of emotional processing, including unprocessed emotions, avoidance, and impoverished emotional expression, although no differences were found in overall emotional processing. Psychopathic traits were positively associated with emotional processing difficulties, with the strongest associations observed for the affective and interpersonal facets. Emotional controllability emerged as the dimension most consistently associated with psychopathy. Regression analyses revealed no evidence that sex moderated the association between psychopathic traits and emotional processing. These findings provide preliminary indicators that psychopathic traits may be associated with emotional processing difficulties in incarcerated individuals and that sex did not significantly moderate these associations in the present sample. The results also support a dimensional, facet-based perspective on emotional dysfunction in psychopathy, although these conclusions should be interpreted with appropriate caution.

## 1. Introduction

Psychopathy is characterized by interpersonal, affective, and behavioral traits, including a shallow emotional responsiveness, lack of empathy and remorse, manipulativeness, impulsivity, aggression, and antisocial behavior (Hare, 2003; Neumann et al., 2007). In the forensic context, psychopathy is of particular relevance because it is more prevalent than in the general community (e.g., Cunha et al., 2025), and due to its strong association with institutional misconduct, poor adjustment to incarceration, and criminal recidivism (Boduszek et al., 2016; Campbell et al., 2009; R. A. Gonçalves, 2004; Guy et al., 2005; Pinheiro et al., 2021; Singh et al., 2011).

The nature of psychopathy has been widely debated, particularly concerning its dimensional versus taxonomic conceptualization (Sellbom & Drislane, 2021). Dimensional approaches conceptualize psychopathy as a continuum of traits, with individuals classified according to the degree and type of phenotypic manifestations of psychopathy (Sellbom & Drislane, 2021), whereas taxometric perspectives define psychopathy as a discrete, naturally occurring category and posits that psychopathic individuals constitute a qualitatively distinct subgroup of individuals who perpetrated crimes (Skilling et al., 2001). From a structural point of view, psychopathy has typically been conceptualized as a multifaceted construct (Edens et al., 2006) comprising four interrelated factors derived from the PCL-R: interpersonal (e.g., superficial charm, grandiosity, manipulation), affective (e.g., shallow affect, lack of empathy, absence of guilt or remorse), lifestyle (e.g., impulsivity, irresponsibility, need for stimulation), and antisocial factors (e.g., poor behavioral control, early conduct problems, criminal versatility) (Hare, 2003; Neumann et al., 2007). Some researchers have further differentiated psychopathy into primary and secondary variants, whereby primary psychopathy is typically characterized by low anxiety, emotional detachment, and pronounced affective deficits (e.g., reduce guilt and empathy), whereas secondary psychopathy is associated with emotional dysregulation, heightened negative affect, impulsivity, and greater susceptibility to environmental and developmental influences (Hicks et al., 2004; Karpman, 1948).

Empirical research demonstrates that elevated levels of psychopathy is robustly associated with maladjustment in prison settings (L. C. Gonçalves et al., 2014; Pinheiro et al., 2021). Prior criminal history has also been identified as a significant risk factor for prison misconduct, with antisocial psychopathic traits contributing to persistence and escalation of disruptive and inappropriate behaviors during incarceration (L. C. Gonçalves et al., 2014; Thomson et al., 2016). Consistently, higher overall levels of psychopathy have been linked to increased rates of institutional rules violations, including both general and violent misconduct (Campbell et al., 2009; Guy et al., 2005; Leistico et al., 2008; Singh et al., 2011). In addition, some evidence suggests that antisocial psychopathic traits and substance abuse may be associated with higher rates of institutional infractions (Pinheiro et al., 2021). More recent evidence suggests that, beyond psychopathic traits, broader personality characteristics and emotional functioning contribute to adaptation within correctional settings, influencing institutional adjustment, aggression, prosocial functioning, and engagement in rehabilitation programs (Hurezan et al., 2026; Rada & Forțu, 2026).

### 1.1. Sex-Specific Manifestations of Psychopathy

Research has highlighted the importance of understanding sex differences in the prevalence, manifestation and underlying mechanisms of psychopathy (De Brito et al., 2021; Verona & Vitale, 2018). Previous reviews have suggested that psychopathy may manifest differently in women, both in terms of prevalence and phenotypic expression, raising important questions regarding the applicability of traditionally male-derived conceptualizations of psychopathy to female populations (Cale & Lilienfeld, 2002; Forouzan & Cooke, 2005). These differences may reflect both sex-related variations in the expression of psychopathic traits and challenges associated with their assessment in women (Cale & Lilienfeld, 2002; Forouzan & Cooke, 2005; Verona & Vitale, 2018).

In addition, the literature consistently indicates that psychopathy is less prevalent in women than in men, both in community and forensic samples (De Brito et al., 2021; Grann, 2000; Pinheiro et al., 2020; Verona & Vitale, 2018). In forensic settings, the prevalence estimates indicate that approximately 10.3% of incarcerated women meet criteria for psychopathy (Guay et al., 2018), compared to rates ranging from 15% to 30% among men (Fox & DeLisi, 2019; Vitale et al., 2002). Across studies employing PCL-based measures (i.e., PCL-R and PCL: SV), women consistently exhibit lower scores on psychopathic traits, with only a small proportion reaching the conventional cut-off score of 30 (Edwards et al., 2019; Pinheiro et al., 2024).

Importantly, although less prevalent, psychopathic traits in women remain predictive of future criminal and violent behavior (Pinheiro et al., 2022), in patterns comparable to those observed in men (Patrick, 2018). In females who perpetrated crimes, sex differences also emerge in the expression of aggression, with women with psychopathic traits displaying less overt physical violence than men (Bailey, 2010). However, they are more likely to engage in manipulative, verbal, relational, and indirect forms of aggression (Bailey, 2010; Lehmann & Ittel, 2012; Pinheiro et al., 2020). Moreover, facet-level analyses reveal sex-specific associations between psychopathy dimensions and aggression, such that the affective facet is linked to physical aggression in women, whereas the antisocial facet is associated with indirect aggression in men (Thomson et al., 2019).

Differences between primary and secondary psychopathy have also been documented, with primary psychopathy appearing more prevalent in men and secondary psychopathy more frequently observed in women (Falkenbach et al., 2017; Moffett et al., 2020). Secondary psychopathy has been associated with externalizing disorders, functional impairment and violent or deviant behavior in institutional settings, among both men and women (Hicks et al., 2004; Skeem et al., 2007). Given that psychopathy encompasses traits directly linked to emotional functioning (Hare, 2003; Neumann et al., 2007; Patrick, 2007), examining emotional processing becomes essential for understanding the affective deficits that underlie its interpersonal and behavioral manifestations (Blair, 2005; Cleckley, 1941; Patrick, 2007).

### 1.2. Psychopathy and Emotional Processing Deficits

Emotional processing refers to the mechanisms through which individuals perceive, interpret, and respond to emotionally salient stimuli, allowing emotional information to guide behavior and social interaction (Gaertner et al., 2021; Gonzalez et al., 2020; Gross & John, 2003; Kret & Ploeger, 2015). Rather than representing a single process, emotional processing encompasses multiple domains of emotional functioning, including emotional recognition, physiological and affective responsiveness, emotional regulation, and emotional expression (Baker et al., 2010; Gross & Jazaieri, 2014; Kret & Ploeger, 2015). Accordingly, impairments in emotional processing may emerge through different mechanisms and affect distinct aspects of emotional functioning (Baker et al., 2010; Gross & Jazaieri, 2014; Snowden et al., 2022). Research examining emotional processing in psychopathy has focused on several of these domains, including fear conditioning, emotional recognition, physiological responsiveness, emotional experience, and emotion regulation (Brook et al., 2013; Patrick, 2007; Verona et al., 2013). These findings suggest that emotional dysfunction in psychopathy may manifest through different emotional processes rather than through a single underlying deficit (Brook et al., 2013; Snowden et al., 2022; Verona et al., 2013).

Psychopathy has traditionally been characterized by a diminished capacity to experience and integrate affective states, compromising the attribution of emotional meaning to events and actions (Patrick, 2007; Pinheiro et al., 2024). Empirical support for this view derives primarily from psychophysiological findings, notably reduced potentiation of the aversive startle reflex, interpreted as evidence of deficient fear responding, a mechanism considered central to psychopathic traits (Patrick, 2007; Pinheiro et al., 2024). This interpretation aligns with the low fear hypothesis, originally proposed by Lykken (1995), which posits that a fearless temperament predisposes individuals to antisocial behavior by weakening behavioral inhibition and disrupting avoidance learning in response to punishment cues (Fowles & Dindo, 2006), thereby impairing the internalization of social norms (Oskarsson et al., 2021). This perspective gains strength in neurocognitive models, which emphasize that deficits in processing signals of emotional distress, especially fear and sadness, hinder normal socialization processes, favoring the development of callous and antisocial traits (Blair, 2005, 2007). However, a critical review and meta-analysis by Hoppenbrouwers et al. (2016) proposes a reassessment of this evidence, differentiating automatic threat processing (visceral and behavioral responses to imminent threats, such as skin conductance, startle reflex, and conditioning) from the conscious experience of fear (subjective negative valence and emotion identification), concluding that the reduction in the subjective experience of fear is much less robust. In other words, this distinction suggests that what has often been interpreted as “reduced fear” or “fearlessness” may reflect impairments in automatic defense mechanisms rather than a global absence of the conscious experience of fear (Hoppenbrouwers et al., 2016).

Nevertheless, evidence indicating that passive avoidance deficits are most pronounced under reward-focused conditions, and attenuated when attention is directed toward avoidance contingencies, challenges the notion of a global fear deficit (Newman & Kosson, 1986). In this sense, the response modulation hypothesis (HMR) proposes that the primary dysfunction in psychopathy reflects impaired attentional shifting rather than an absence of fear per se (Newman et al., 2010). Specifically, individuals with psychopathic traits demonstrate difficulty reallocating attention from dominant goal-directed activity to peripheral yet motivationally relevant cues, including threat signals, which may account for context-dependent reductions in fear responsiveness (Hamilton & Newman, 2018).

Classical theories further propose that psychopathy involves impaired anticipation of negative consequences, reflected in deficient anticipatory fear and weakened behavioral inhibition (Hare, 1978). This dysfunction has been linked to disrupted associative learning between external threat cues and internal affective responses, limiting the capacity to learn adaptively from punishment (Patrick, 1994). Neurobiological evidence supports this perspective, with studies reporting atypical activation in cortical and subcortical regions involved in emotional learning and regulation, including the dorsolateral prefrontal cortex and the amygdala, during emotional conditioning tasks (Pinheiro et al., 2024).

Beyond fear conditioning, psychopathy has been consistently associated with specific deficits in emotional recognition and responsiveness (Dolan & Fullam, 2006; Iria & Barbosa, 2009). Individuals with psychopathic traits show impairments in non-verbal emotional processing, particularly in the recognition of facial expressions of fear and sadness (Dolan & Fullam, 2006; Iria & Barbosa, 2009), accompanied by reduced sensitivity to others’ emotional distress and attenuated physiological responses to stimuli depicting suffering (Fullam & Dolan, 2006; Łuczak & Tanaś, 2021). Such impairments have been interpreted as reflecting a broader deficit in affective information processing (Brook et al., 2013).

Empirical evidence also indicates that emotional processing deficits vary across psychopathy dimensions (Snowden et al., 2022). The affective–interpersonal factor has been primarily associated with reduced empathy, shallow affect, and attenuated physiological reactivity to unpleasant stimuli, whereas impairments in processing negatively valenced emotions, such as fear and anger, have been more closely linked to the antisocial factor (Edwards et al., 2019; Hare & Neumann, 2008; Snowden et al., 2022; Verona et al., 2013).

Overall, converging evidence indicates that psychopathy is associated with impairments across multiple domains of emotional functioning, including emotional experience, recognition, regulation, and physiological responsiveness (Brook et al., 2013; Fullam & Dolan, 2006; Patrick, 2007). These difficulties may manifest through distinct mechanisms, ranging from reduced sensitivity to emotional cues and attenuated affective responses to problems in regulating, integrating, and expressing emotional experiences (Baker et al., 2010; Gross & Jazaieri, 2014). These deficits impair sensitivity to others’ emotions and undermine the emotional mechanisms that normally inhibit antisocial and aggressive behavior, providing a key explanatory framework for the interpersonal and behavioral traits of psychopathy (Brook et al., 2013; Fullam & Dolan, 2006).

### 1.3. Sex-Specific Differences in Emotional Processing and Psychopathy

Emotional processing is shaped by biological, neural, and sociocultural factors that influence how emotional stimuli are perceived, evaluated, and expressed (Kret & De Gelder, 2012; Whittle et al., 2011). Neurofunctional research indicates that women generally show stronger neural activation in response to negative and socially salient stimuli, particularly within limbic and prefrontal regions such as the amygdala and prefrontal cortex, whereas men tend to exhibit heightened reactivity to positively valenced and reward-related stimuli (Fine et al., 2009; Whittle et al., 2011). At the behavioral level, women typically rate negative emotional stimuli as more intense and unpleasant and demonstrate superior recall for such information, while men show greater sensitivity to arousal- and reward-related cues (Schienle et al., 2005; van Wingen et al., 2011).

Women tend to express emotions more frequently through facial and verbal channels, whereas men are more likely to externalize emotional states through action-oriented behaviors, including aggression (Kret & De Gelder, 2012). Within psychopathy, dysfunctions in these systems are thought to underlie reduced sensitivity to aversive emotional cues, impaired empathy, and deficits in emotional regulation, although their behavioral expression may differ by sex (Patrick et al., 2009; Verona & Vitale, 2018).

Experimental and psychophysiological studies suggest that women with psychopathic traits may display partially distinct emotional processing profiles compared to men (Verona et al., 2013; Pinheiro et al., 2020). Specifically, women with elevated psychopathic traits scores appear to retain a greater capacity to allocate attention to emotionally salient information, particularly when such information is relevant to goal-directed behavior (Anton et al., 2018). However, women with higher propensities for psychopathy may show blunted arousal to neutral stimuli alongside selective increases in arousal to specific threat-related cues (Pinheiro et al., 2025). Evidence indicates reduced startle potentiation in response to others’ distress among women with higher psychopathic traits, pointing to diminished affective reactivity despite intact perceptual processing (Verona et al., 2013). In contrast, men with psychopathic traits more consistently exhibit pronounced deficits in both attentional and affective components of emotional processing, including blunted physiological responses to aversive stimuli (Justus & Finn, 2007).

Finally, sociocultural factors appear to shape how emotional processing deficits associated with psychopathy are behaviorally manifested (Beryl et al., 2014; de Vogel & Lancel, 2016; Pinheiro et al., 2020). Compared to men, women are more likely to engage in relational, indirect, and covert forms of aggression, including emotional manipulation, rather than overt physical violence (Beryl et al., 2014; de Vogel & Lancel, 2016; Wynn et al., 2012). Although emotional manipulation is observed in both sexes, psychopathy in women is more frequently associated with emotional dysregulation, interpersonal instability, and reactive patterns of affective expression, whereas psychopathy in men is more often characterized by emotional detachment and instrumental aggression (Grieve & Mahar, 2010; Verona & Vitale, 2006).

Taken together, the existing literature highlights sex-related differences in the emotional processing profiles associated with psychopathy, although the nature and extent of these differences remain insufficiently understood.

### 1.4. Current Study

Despite the extensive literature linking psychopathy to emotional processing deficits, research examining sex-related differences in emotional functioning among incarcerated individuals with elevated psychopathic traits remains limited, particularly among females who perpetrated crimes (Pinheiro et al., 2020; Verona et al., 2013). Although psychopathy has been consistently associated with impairments in emotional experience, regulation, and responsiveness to aversive stimuli, accumulating evidence suggests that these deficits may not manifest uniformly across sexes (Brook et al., 2013; Verona & Vitale, 2018; Patrick, 2007; Pinheiro et al., 2024). However, empirical studies directly comparing emotional processing profiles between incarcerated men and women with elevated psychopathic traits remain scarce, particularly within the Portuguese forensic context (Pinheiro et al., 2020; Pinheiro et al., 2024).

Emotional processing has been identified as a fundamental mechanism underlying the affective and interpersonal characteristics of psychopathy (Patrick, 2007). Research has demonstrated its critical involvement in aggressive behavior, institutional misconduct, and adjustment to incarceration (Campbell et al., 2009; L. C. Gonçalves et al., 2014; Pinheiro et al., 2021). Building on prior evidence suggesting that women with psychopathic traits may retain certain attentional capacities toward emotional information while still exhibiting affective blunting, a sex-specific examination of emotional processing dimensions is particularly warranted (Verona et al., 2013). In the present study, emotional processing was operationalized as self-reported difficulties in the regulation, integration, and expression of emotional experiences, consistent with contemporary conceptualizations of the construct (Baker et al., 2010; Gross & Jazaieri, 2014; Kret & Ploeger, 2015).

Accordingly, the primary objective of the present study is to examine the associations between psychopathic traits (total score and factor-level dimensions) and emotional processing in incarcerated individuals. Specifically, the study aims to compare psychopathy scores and emotional processing between incarcerated men and women; examine the association between psychopathic traits and emotional processing; test whether sex moderates the association between psychopathic traits and emotional processing; and identify which psychopathy facets are most strongly associated with emotional processing difficulties.

By including both male and female participants and by modeling a dimensional approach, the present study seeks to clarify whether emotional processing impairments associated with psychopathy are better explained by core psychopathic traits and whether these associations differ by sex within incarcerated populations (Hare, 2003; Sellbom & Drislane, 2021).

Based on existing literature, the following hypotheses were formulated:

**H1.**  *Men were expected to report higher psychopathy scores than women, particularly in the lifestyle and antisocial facets (Verona & Vitale, 2018).*

**H2.**  *Women were expected to report greater emotional processing difficulties, particularly in dimensions related to emotional expression and unprocessed emotions (Verona & Vitale, 2018).*

**H3.**  *Higher levels of psychopathic traits (total score) were expected to be associated with greater emotional processing difficulties (Brook et al., 2013; Patrick, 2007).*

**H4.**  *It was expected that the affective and interpersonal facets would show the strongest associations with emotional processing difficulties (Hare, 2003; Patrick, 2007).*

It was also examined whether sex moderates the association between psychopathic traits and emotional processing (i.e., Sex × Psychopathy interactions). Based on prior literature suggesting potential sex-specific emotional profiles (Verona et al., 2013), it was expected that the strength of the association might differ between men and women; however, given inconsistent evidence in the literature, these analyses were treated as hypothesis-driven but exploratory.

This research helps address existing gaps in the literature, particularly regarding females who perpetrated crimes, and offers empirical insights with potential implications for assessment and intervention in forensic settings (Pinheiro et al., 2020; Verona et al., 2013).

## 2. Materials and Methods

### 2.1. Participants

The sample comprised 257 incarcerated individuals, including 144 (56%) men and 113 (44%) women, who were serving prison sentences or were in pretrial detention in prisons in the northern and central regions of Portugal, all aged 18 or over. Most participants were Portuguese (*n* = 232; 90.3%), with a mean age of 40.71 years (*SD* = 11.23), ranging from 20 to 81 years. More than half of participants were single (*n* = 140; 54.5%) and had completed between nine and 12 years of education (*n* = 140; 54.5%). The majority of the sample had been sentenced (*n* = 213; 82.9%), with a mean sentence length of 95.49 months (*SD* = 74.27). A total of 160 (62.3%) participants were sentenced for the first time.

### 2.2. Instruments

#### 2.2.1. Sociodemographic and Socio-Legal Questionnaire

A questionnaire was developed to gather data on age, marital status, education level, current legal status (e.g., type of sentence; length of sentence), and criminal history to characterize the sample. This information was collected through interviews with individuals in prison.

#### 2.2.2. Self-Report Psychopathy-Short Form (SRP-SF)

The Self-Report Psychopathy-Short Form (SRP-SF) is a 29-item self-report measure designed to assess psychopathic traits (Seara-Cardoso et al., 2019), derived from the 64-item complete version of the SRP-4 (Hare, 1980). The instrument follows the same factor structure as the PCL-R, assessing four facets of psychopathy: the interpersonal (INT) factor assesses covert antisocial characteristics, such as pathological lying and manipulation; the affective factor (AFF) explores the affective aspects of psychopathy, such as difficulty feeling empathy and lack of guilt or concern for others; the lifestyle (LIF) factor examines impulsive and reckless behavior; and the antisocial factor (ANT) explores overt antisocial behavior (Seara-Cardoso et al., 2019). Items are rated on a five-point Likert scale (1 = strongly disagree to 5 = strongly agree), with higher scores indicating greater levels of psychopathic traits. In the Portuguese validation, the scale demonstrated a good fit for the four-factor structure, which was supported in both male and female subsamples, as well as adequate psychometric properties across gender groups. The model also showed good internal consistency (*α* = 0.87) and expected patterns of associations with external correlates of psychopathy (Seara-Cardoso et al., 2019). In the present study, Cronbach’s alpha coefficients ranged from 0.56 (AFF), indicating relatively low internal consistency, to 0.77 (INT), indicating acceptable internal consistency. The total SRP-SF score demonstrated excellent internal consistency (*α* = 0.91).

#### 2.2.3. The Emotional Processing Scale (EPS-25)

The Emotional Processing Scale (EPS-25) is a self-report measure designed to assess emotional processing styles and deficits (Baker et al., 2010). The EPS-25 is structured into 25 items and five dimensions: the avoidance enables the analysis of the experiential or internal avoidance of emotional stimuli; the suppression allows the evaluation of the suppression of negative emotional states and their expression; the controllability considers the inability to control intense, externally-oriented negative emotions; the impoverished emotional experience identifies impoverished emotional experiences; and signs of unprocessed emotion assesses unprocessed emotion signals (Baker et al., 2010; Lauriola et al., 2021). Each dimension consists of five items, which are answered on a graded scale ranging (0 = strongly disagree to 9 = strongly agree). The scale’s internal consistency is high, with a Cronbach’s alpha value of 0.92, as well as strong test–retest reliability (Baker et al., 2010). The exploratory factor analysis revealed a five-factor solution, accounting for 59.4% of the total variance. In this study, we obtained an alpha for the total scale of 0.93. Cronbach’s alphas were also calculated for the different subscales: suppression = 0.79, signs of unprocessed emotion = 0.82, controllability = 0.81, avoidance = 0.67, and impoverished emotional experience = 0.80.

### 2.3. Procedure

The present study was approved by the University of Minho Research Ethics Committee in Social and Human Sciences. All procedures were conducted in accordance with the ethical standards established in the Declaration of Helsinki and its subsequent amendments, ensuring respect for participants’ dignity, autonomy, confidentiality, and right to withdraw without penalty. Following ethical approval, formal authorization to conduct data collection within correctional facilities was obtained from the General Directorate of Reintegration and Prison Services—Ministry of Justice (DGRSP-MJ). After institutional approval was granted, the study was disseminated across the participating prison settings.

Potential participants were identified among incarcerated individuals in different institutional contexts within the prison facilities, namely those engaged in work activities, attending school, or resting in their cells. From this pool, individuals were randomly selected and subsequently invited to attend an information session about the study. The selected participants were directed to the prison schoolrooms, which were considered the most appropriate and spaces available in the institutions where the research took place. During the session, members of the research team met with interested individuals to provide a detailed explanation of the study’s objectives, procedures, potential risks, and safeguards. Particular emphasis was placed on the voluntary nature of participation, the absence of any legal or institutional consequences associated with refusal or withdrawal, and the confidentiality and anonymity of the collected data. Written informed consent was obtained from all participants prior to data collection. Consent forms clearly described the purpose of the research, data handling procedures, confidentiality measures, and participants’ rights. Following the provision of informed consent, individual interviews were conducted with each participant to collect relevant sociodemographic and legal–penal information. Subsequently, participants completed the self-report instruments included in the research protocol. All collected data were coded using numerical identifiers and stored separately from any directly identifying information.

### 2.4. Data Analysis

Data were analyzed using SPSS Statistics 29.0 software. Prior to conducting the main analyses, all relevant statistical assumptions were examined. For regression analyses, assumptions of linearity, normality of residuals, homoscedasticity, and absence of multicollinearity were assessed through visual inspection of residual plots, normal probability plots, and variance inflation factor (VIF) values. No major violations were detected that would compromise the validity of the analyses. With regard to missing data, analyses were conducted using a complete-case approach, whereby only participants with valid data on the SRP-SF and EPS-25 were included.

To characterize the sample, descriptive analyses of socio-demographic and legal variables were carried out using means, standard deviations, and frequencies. Sex differences in categorical variables, including marital status, nationality, educational level, legal status, re-offense status, and psychopathy propensity category, were examined using Pearson’s chi-square tests. Effect sizes were estimated using Cramer’s *V*. For continuous variables, including age and sentence length, independent samples *t*-tests were performed. Cohen’s *d* values are reported as absolute values to reflect effect size magnitude, with the direction of differences interpreted based on group means and the sign of the *t*-statistics. Effect sizes were interpreted according to conventional benchmarks (small = 0.20, medium = 0.50, large = 0.80; Cohen, 1988). To examine sex differences in psychopathic traits and emotional processing, ANCOVA analyses controlling for age were conducted comparing men and women on total and factor scores of the SRP-SF and the EPS-25. Effect sizes were reported using partial eta squared (η^2^_p_) and interpreted according to conventional benchmarks (0.01 = small, 0.06 = medium, 0.14 = large). To account for multiple comparisons, *p*-values were adjusted using the Benjamini–Hochberg (B-H) procedure to control the false discovery rate. Results indicated that all effects with *p* ≤ 0.024 remained statistically significant following correction.

Pearson correlation analyses were performed to analyze associations between psychopathy (total scores and facets) and emotional processing (total scores and dimensions).

Hierarchical multiple regression analyses were conducted to examine the potential moderating role of sex. Emotional processing (EPS total score) was entered as the dependent variable in all models. In the first step (Model 1), covariates were entered, including age, nationality, educational level, type of offense (single vs. multiple crimes), and sex. In the second step (Model 2), psychopathy was entered as a predictor, operationalized either as the SRP-SF total score or as individual facet scores (interpersonal, affective, lifestyle, and antisocial). In the third step (Model 3), interaction terms between sex and psychopathy (Sex × Psychopathy) were included to test for moderation effects and to formally examine whether the associations between psychopathic traits and emotional processing differed by sex. Separate regression models were conducted for each psychopathy facet to avoid multicollinearity and to allow for a clearer interpretation of facet-specific effects. Model fit was evaluated using R^2^ and changes in explained variance (Δ*R*^2^) across steps. The statistical significance of changes in model fit was assessed using *F*-change tests. Regression coefficients (unstandardized B, standard errors, standardized *β*, *t* values, and *p* values) were examined to interpret the direction and strength of associations.

## 3. Results

### 3.1. Sex Differences in Sociodemographic Variables

Significant sex differences were observed across several sociodemographic and legal variables (cf. Table 1). Women were significantly older than men, *t* = 2.583, *p* = 0.010, *d* = 0.574, indicating a moderate effect size. Significant sex differences emerged in nationality, *χ*^2^ = 11.533, *p* < 0.001, *V* = 0.212. Women were proportionally more likely to have a non-Portuguese nationality compared to men. Educational level also differed significantly between sexes, *χ*^2^ = 29.885, *p* < 0.001, *V* = 0.341, with women exhibiting higher rates of lower educational attainment, particularly at the 4th-grade level, whereas men were more frequently represented in the 6th- and 9th-grade categories.

A significant difference was also found in re-offense status, *χ*^2^ = 12.523, *p* < 0.001, *V* = 0.221. Men were more likely to commit crimes repeatedly, whereas women were more often convicted for the first time.

### 3.2. Sex Differences in Psychopathy and Emotional Processing

ANCOVA analyses controlling for age were conducted to examine sex differences in psychopathic traits and emotional processing (see Table 2).

Significant sex differences emerged for total psychopathy scores, with men scoring significantly higher on the SRP-SF total score compared to women, *F* = 18.557, *p* < 0.001, η^2^_p_ = 0.128. At the facet level, men also scored significantly higher than women across all psychopathy dimensions (see Table 2), with effect sizes ranging from medium to large. All reported effects remained statistically significant after BH correction (*p* ≤ 0.024).

Regarding emotional processing, no significant sex differences were found in the EPS-25 total score, *F* = 2.518, *p* = 0.083, η^2^_p_ = 0.020. At the subscale level, women scored significantly higher than men on signs of unprocessed emotions, *F* = 3.843, *p* = 0.023, η^2^_p_ = 0.029; avoidance, *F* = 3.779, *p* = 0.024, η^2^_p_ = 0.029; and impoverished emotional expression, *F* = 4.338, *p* = 0.014, η^2^_p_ = 0.034. These subscale effects met the B-H-corrected significant threshold (*p* ≤ 0.024).

### 3.3. Associations Between Emotional Processing and Psychopathic Traits

Pearson correlation analyses were conducted to examine the associations between psychopathy (total and factor scores) and emotional processing in the total sample and separated by sex (see Table 3).

In the total sample, psychopathy total scores were positively associated with overall emotional processing difficulties, although the magnitude of this association was small. At the facet level, significant positive associations with overall emotional processing were observed for the affective, interpersonal, and lifestyle facets. In contrast, the antisocial facet was not significantly associated with overall emotional processing. At the level of specific emotional processing dimensions, the most consistent pattern of associations emerged for emotional controllability. Significant associations with impoverished emotional expression were observed only for the affective facet, whereas psychopathy total scores and the remaining facets were not significantly related to this dimension.

In the male sample, psychopathy total scores were not significantly correlated with the overall emotional processing score. However, significant positive associations emerged in the controllability dimension of emotional processing. At the facet level, the most consistent associations with emotional processing were found for the affective and interpersonal facets. In contrast, the lifestyle and antisocial facets showed weaker and largely non-significant correlations with impoverished emotional expression dimensions.

In the female sample, psychopathy total scores were significantly associated with overall emotional processing difficulties. At the facet level, all dimensions were positively associated with emotional processing, with the affective facet showing the strongest associations. Regarding specific emotional processing dimensions, the most consistent and strongest associations were observed with emotional controllability, across all psychopathy dimensions). Significant positive associations were also found for unprocessed emotional signals (particularly for the affective facet) and impoverished emotional expression. In contrast, associations with emotional suppression were weak and inconsistent, and emotional avoidance was not significantly associated with psychopathy.

### 3.4. Sex as a Moderator of the Association Between Psychopathic Traits and Emotional Processing

A hierarchical regression analysis was conducted to examine whether psychopathic traits (total scores) predicted emotional processing (EPS total score) and whether this association was moderated by sex (cf. Table 4). Model 1 included age, nationality, education, crime type, and sex as covariates; Model 2 added total psychopathy scores (or in separate analyses, psychopathy facets); and Model 3 further included the interaction term between psychopathy (or psychopathy facet) and sex (psychopathy × sex).

Model 1 including covariates (age, nationality, education, and crime type) and sex was significant, *F*(5, 247) = 3.47, *p* = 0.005, explaining 6.6% of the variance in emotional processing. Adding psychopathy (total score) (Model 2) significantly improved model fit, Δ*R*^2^ = 0.047, *p* < 0.001, with higher psychopathy scores predicting greater emotional processing difficulties, *β* = 0.253, *p* < 0.001. However, the interaction between sex and psychopathy (Model 3) was not significant, *β* = 0.115, *p* = 0.253, and its inclusion did not significantly increase explained variance, Δ*R*^2^ = 0.005, *p* = 0.253.

To examine facet-specific effects, separate regression models were conducted (cf. Table 4).

The interpersonal facet (Model 2) significantly predicted emotional processing, *β* = 0.227, *p* < 0.001, explaining an additional 4.4% of variance. However, the interaction (Model 3) with sex was not significant, *β* = 0.041, *p* = 0.662.

The affective facet (Model 2) was the strongest predictor, significantly improving model fit, Δ*R*^2^ = 0.068, *p* < 0.001. Higher affective traits were associated with greater emotional processing difficulties, *β* = 0.278, *p* < 0.001, and this effect remained significant in the final model (Model 3), *β* = 0.212, *p* = 0.033. The interaction with sex was not significant, *β* = 0.085, *p* = 0.381.

The lifestyle facet significantly predicted emotional processing in Model 2, *β* = 0.245, *p* < 0.001, but this effect was not retained in the final model (Model 3), *β* = 0.147, *p* = 0.172. The interaction with sex was also not significant, *β* = 0.123, *p* = 0.216.

The antisocial facet (Model 2) did not significantly predict emotional processing, *β* = 0.086, *p* = 0.221, nor did it interact with sex (Model 3), *β* = 0.152, *p* = 0.173.

## 4. Discussion

The present study aimed to examine the associations between psychopathic traits and emotional processing in incarcerated individuals, compare psychopathy scores and emotional processing between incarcerated men and women, examine the association between psychopathic traits and emotional processing, and test whether sex moderates the association between psychopathic traits and emotional processing. Overall, the findings suggest that, although incarcerated men and women differ in the expression of psychopathic traits, psychopathy is associated with emotional processing difficulties, with no evidence that these associations vary by sex.

### 4.1. Sex Differences in Psychopathic Traits and Emotional Processing

In this study, significant sex differences were observed across psychopathy scores. Men reported higher overall psychopathy as well as higher scores on its constituent factors, consistent with extensive evidence indicating a greater prevalence of psychopathic traits among men, particularly in forensic populations (Cunha et al., 2025; Grann, 2000; Hare, 2003; Verona & Vitale, 2018; Pinheiro et al., 2020). These differences were especially pronounced in antisocial and lifestyle dimensions, supporting prior findings that link male psychopathy to behavioral dysregulation, criminal versatility, and institutional misconduct (R. A. Gonçalves, 2004; Guy et al., 2005; Hare, 2003). This pattern suggests that psychopathy in men may be more expressed through externalizing and behaviorally observed traits (Verona & Vitale, 2018). In contrast, women presented lower overall psychopathy scores, which should not be interpreted as reduced clinical relevance. Rather, psychopathy in women may be less overt and more reflected in interpersonal and affective characteristics, which may not be fully captured by global scores (Pinheiro et al., 2020). This distinction may also account for the smaller sex differences observed in some psychopathy dimensions, highlighting potentially different manifestations shaped by developmental and sociocultural factors (Beryl et al., 2014; de Vogel & Lancel, 2016; Pinheiro et al., 2020).

Regarding emotional processing, no sex differences were found at the global level; however, women reported greater difficulties in specific domains, namely unprocessed emotions, avoidance, and impoverished emotional expression. These findings align with previous research indicating higher levels of emotional distress and dysregulation among women in forensic settings (Verona & Vitale, 2006, 2018). Elevated avoidance scores suggest difficulties in integrating emotional experiences, while higher impoverished emotional expression indicates challenges in translating internal states into adaptive interpersonal communication (Baker et al., 2010).

These patterns may be understood within the broader context of adversity and trauma. Justice-involved women are disproportionately exposed to cumulative interpersonal victimization, including childhood abuse and intimate partner violence (de Vogel & Nicholls, 2016; Sousa et al., 2025). Such experiences are known to impair emotional development, compromise emotional regulation capacities, and increase vulnerability to affective dysregulation throughout adulthood (Cloitre et al., 2009; Ford & Courtois, 2014; Sousa et al., 2026), and may contribute to the emotional processing difficulties observed in this study. From this perspective, these difficulties likely reflect not only psychopathic traits but also trauma-related adaptations that shape emotional functioning (Pinheiro et al., 2025).

Overall, the findings suggest that sex differences are more evident in the expression of psychopathy, with men showing more behavioral manifestations and women exhibiting more nuanced emotional and interpersonal patterns. Emotional processing difficulties among women appear to be primarily related to challenges in integrating and expressing emotions, rather than global deficits in emotional processing.

### 4.2. Associations Between Psychopathic Traits and Emotional Processing

In the total sample, psychopathic traits were positive but modestly associated with emotional processing difficulties, suggesting that emotional dysfunction in psychopathy is not generalized but rather confined to specific domains. Consistent with this interpretation, the most robust associations emerged for emotional controllability, which was moderately associated with total psychopathy and all facets. This dimension reflects difficulties in managing and regulating emotional responses under heightened arousal (Baker et al., 2010) and aligns with theoretical models emphasizing impairments in regulation, inhibition, and impulse control as central features of psychopathy (Hare, 2003; Patrick, 2007). In correctional contexts, such difficulties may contribute to maladaptive responses, including impulsivity and reactive aggression (Campbell et al., 2009; R. A. Gonçalves, 2004).

At the facet level, the affective dimension showed the strongest and most consistent associations, particularly with emotional controllability and emotional expression. These results are theoretically coherent, given that affective traits (e.g., callousness, shallow affect) reflect core emotional deficits (Hare, 2003; Patrick, 2007). This pattern is also consistent with neurocognitive models proposing that psychopathy is characterized by atypical processing of emotionally salient information, particularly cues related to distress, fear, and interpersonal suffering (Blair, 2005). Consequently, emotional dysfunction may represent a core mechanism underlying psychopathic traits rather than merely a consequence of maladaptive behavior. The findings therefore support models positing that emotional processing impairments in psychopathy are primarily rooted in these affective characteristics, rather than in behavioral manifestations alone (Skeem & Cooke, 2010). In contrast, the interpersonal and lifestyle facets demonstrated weaker and more circumscribed associations, mainly linked to emotional controllability. This pattern suggests that traits such as manipulativeness, superficial charm, impulsivity, and irresponsibility may contribute to difficulties in emotion regulation, but do not appear to be strongly associated with broader emotional processing impairments. This differentiation aligns with the distinction proposed by Patrick et al. (2009) between boldness—an interpersonal facet, frequently associated with emotional resilience and social dominance—and disinhibition—related to the lifestyle facet—dimensions that do not necessarily imply the same core affective deficits. Similarly, the antisocial facet showed minimal associations with emotional processing, reinforcing the distinction between core emotional traits and their behavioral manifestations.

From a contemporary perspective, antisocial behavior is better understood as arising from the interaction between dispositional traits and criminogenic cognitions, such as cognitive distortions, offense-supportive beliefs, and moral disengagement (Bonta & Andrews, 2023; Walters, 2019). Within this framework, affective and interpersonal deficits may contribute to maladaptive cognitive schemas, but do not directly translate into behavior. Instead, antisocial conduct appears more proximally driven by these cognitive processes. Accordingly, the findings suggest that emotional dysfunction is primarily linked to affective traits, whereas antisocial behavior reflects a downstream outcome shaped by cognitive–affective interactions, rather than emotional deficits alone (Hare, 2003; Patrick, 2007). This interpretation is consistent with recent integrative models emphasizing the role of dynamic cognitive processes in the enactment of offending behavior (Serin et al., 2016; Willis & Ward, 2024).

When examining sex-specific patterns, the overall structure of associations between psychopathy and emotional processing was similar for men and women, although differences in magnitude emerged. In men, associations were more limited and primarily restricted to emotional controllability, suggesting a more circumscribed link between psychopathy and emotional regulation. This pattern is consistent with profiles characterized by reduced emotional responsiveness and regulatory control (Hare, 2003; Patrick, 2007; Neumann et al., 2007). This finding aligns with studies indicating that psychopathy in men is more frequently expressed through emotional detachment and deficits in both affective responsiveness and regulatory control (Verona & Vitale, 2018). In contrast, women showed broader and more consistent associations across psychopathy facets and emotional processing domains, including emotional expression and unprocessed emotions. This pattern could suggest that, among incarcerated women, psychopathic traits might be associated with more pervasive emotional processing difficulties, potentially extending beyond regulatory impairments. Importantly, although women presented lower overall psychopathy scores, psychopathic traits were more consistently associated with emotional processing difficulties, pointing to a more complex emotional profile rather than simple emotional detachment (de Vogel & Nicholls, 2016; Verona & Vitale, 2018). These differences may be understood in light of the higher prevalence of trauma and emotional distress among justice-involved women (Verona & Vitale, 2006, 2018; Pinheiro et al., 2020), which can shape emotional regulation and processing (Verona et al., 2013; Pinheiro et al., 2020).

However, despite differences in magnitude, the overall pattern of findings does not support fundamentally distinct mechanisms linking psychopathic traits to emotional processing across sexes. Rather, the results suggest that although women may report higher levels of emotional difficulties, the association between psychopathic traits and emotional processing remains structurally similar. This interpretation is supported by evidence demonstrating substantial structural similarity in psychopathy across sexes, including comparable factor structures and patterns of association with external correlates (Neumann et al., 2012; Verona et al., 2013). Consequently, the present findings lend further support to the view that sex differences in psychopathy primarily reflect variations in expression and severity, rather than as qualitatively distinct etiological processes (Verona et al., 2013; Sellbom & Drislane, 2021).

Taken together, the results suggest that emotional dysfunction in psychopathy is not characterized by a global impairment, but rather by more specific deficits in emotional regulation and processing, particularly in the management of emotional responses and, in the case of affective traits, in emotional experience and expression. This is consistent with theoretical models emphasizing impairments in attentional processes, associative learning, and affective responding (Newman et al., 2010; Patrick, 2007), as well as with empirical work documenting reduced emotional recognition and responsiveness, particularly for aversive or distress-related stimuli (Dolan & Fullam, 2006; Brook et al., 2013). Accordingly, emotional dysfunction in psychopathy seems to be more accurately conceptualized as reflecting atypical patterns of emotional processing—such as difficulties attending to, integrating, and regulating emotional information—rather than a simple absence of emotional experience.

### 4.3. The Role of Sex as a Moderator

Consistent with this perspective, regression analyses examining the moderating role of sex indicated that the association between psychopathic traits and emotional processing does not vary as a function of sex. This finding challenges assumptions of sex-specific pathways linking psychopathy to emotional dysfunction in incarcerated populations. Although prior research has suggested that men and women with psychopathic traits may exhibit distinct emotional processing profiles, such as relatively preserved attentional processing in women despite affective deficits (Verona et al., 2013; Pinheiro et al., 2020), such conclusions have often relied on subgroup comparisons rather than formal tests of interaction. By directly modeling sex as a moderator, the present study provides more rigorous evidence that the structural relationship between psychopathic traits and emotional processing is comparable across men and women. Although descriptive differences in emotional processing were observed, consistent with research indicating higher levels of emotional distress and affective dysregulation among incarcerated women (Verona & Vitale, 2006, 2018), these did not translate into differential associations with psychopathy. This distinction is important, as it suggests that while men and women may differ in overall levels of emotional difficulties, the mechanisms linking psychopathic traits to these difficulties appear to be shared.

From a theoretical standpoint, these findings support models conceptualizing psychopathy as involving core deficits in emotional processing and regulation that are not inherently sex-specific (Hare, 2003; Patrick, 2007). In particular, the absence of moderation effects aligns with perspectives emphasizing deficits in affective processing, attentional modulation, and regulatory control as central traits of psychopathy (Newman et al., 2010). These mechanisms appear to operate similarly across sexes, even if their behavioral expression may differ. Finally, these results underscore the importance of using interaction-based analytical approaches when examining sex differences. Apparent differences observed in sex-stratified analyses may reflect variations in baseline levels rather than true differences in the nature or strength of associations. Without formally testing moderation, there is a risk of overinterpreting sex-specific effects.

In sum, the absence of significant interaction effects indicates that psychopathy is associated with emotional processing difficulties in a consistent manner across men and women, supporting a dimensional and sex-comparable understanding of emotional dysfunction in psychopathy.

### 4.4. Limitations and Clinical and Forensic Implications

Although this study offers important contributions, several limitations should be considered. Both psychopathy and emotional processing were assessed using self-report measures (SRP-SF and EPS-25), which may introduce biases such as social desirability, recall inaccuracies, and limited emotional insight—particularly in forensic populations, where individuals with elevated psychopathic traits may engage in impression management (Hare, 2003; Patrick, 2007). To address these limitations, future research should combine self-report instruments with clinician-rated assessments, interviews, or physiological measures to strengthen validity. Although overall internal consistency was acceptable, some subscales showed lower reliability, namely the EPS-25 avoidance dimension and the SRP-SF affective and antisocial facets. This may be partly explained by the relatively small number of items in certain subscales (Baker et al., 2010), as well as by the complexity and variability of the underlying constructs across individuals and forensic contexts (R. A. Gonçalves, 2004; Verona & Vitale, 2018) and should be considered when interpreting facet-level results. Furthermore, the data were collected exclusively from incarcerated individuals in prisons located in the northern and central regions of Portugal, which may limit the generalizability of the findings to other penitentiary populations, such as individuals in different security levels, pretrial detainees, community-based correctional settings, or inmates from other cultural and institutional contexts. Future studies should therefore aim to include more diverse and representative samples, as well as cross-national comparisons. Finally, further validation of emotional processing measures in forensic settings would contribute to improving the robustness of future findings (Hare, 2003; Sellbom & Drislane, 2021).

Despite the limitations, the present findings offer relevant clinical and forensic implications. Overall, the results suggest that associations between psychopathic traits and emotional processing are not uniform, but appear to vary across dimensions, with more consistent links observed for affective and, to a lesser extent, interpersonal traits. In contrast, lifestyle and antisocial traits showed weaker or non-significant associations. These patterns support the value of a facet-based approach when examining emotional functioning in individuals with psychopathic traits. In particular, the observed associations involving affective traits—especially with emotional controllability—may indicate that interventions targeting emotional awareness, affect regulation, and the processing of emotional information could be relevant for individuals with elevated affective features (Patrick, 2007), although such implications should be interpreted with appropriate caution.

Regarding sex, the absence of a statistically significant moderation effect suggests that the association between psychopathy and emotional processing did not differ between men and women in this sample, rather than providing evidence of equivalence across groups. Although women reported higher levels of emotional difficulties at a descriptive level, these differences did not translate into differential associations with psychopathic traits. This may indicate that similar underlying mechanisms are involved; however, conclusions about sex-specific processes should remain tentative. At the same time, baseline differences in psychopathic traits and emotional functioning may still be clinically relevant and support the use of sex-sensitive assessment and intervention approaches (Pinheiro et al., 2020; Verona & Vitale, 2018). Greater emotional distress among women may suggest the potential benefit of incorporating components addressing emotional awareness and regulation (Sousa et al., 2025; Verona & Vitale, 2006, 2018). Importantly, intervention approaches in correctional settings should not focus exclusively on personality traits or psychopathy, but also consider criminogenic cognitions, which are known to play a central role in offending behavior and institutional adjustment (Bonta & Andrews, 2023). Integrating both personality-related and cognitive factors may therefore enhance the effectiveness of rehabilitation strategies.

Finally, the findings support the potential relevance of including emotional processing in forensic assessment and intervention planning. Emotional processing difficulties may be one component associated with psychopathic traits, with possible implications for empathy, behavioral regulation, and responsiveness to emotional cues (Patrick, 2007). Incorporating these aspects into assessment frameworks may contribute to more comprehensive and individualized approaches, while acknowledging that they represent only one element within a broader set of risk and rehabilitation factors.

## 5. Conclusions

The present study contributes to the understanding of the relationship between psychopathic traits and emotional processing in an incarcerated population by adopting a dimensional, facet-based approach. By examining these constructs in both men and women, it provides a more comprehensive view of how emotional functioning relates to psychopathic traits.

The findings indicate that associations between psychopathic traits and emotional processing are not uniform, but vary across dimensions, with more consistent links observed for affective and, to a lesser extent, interpersonal traits. In contrast, lifestyle and antisocial traits showed weaker or non-significant associations. These results support the importance of considering the multidimensional nature of psychopathy when examining emotional functioning. Although men and women differed in baseline levels of psychopathic traits and emotional processing, no evidence was found that sex moderated the relationship between these variables. This suggests that the associations observed in this study do not differ significantly between sexes, although conclusions regarding underlying mechanisms should remain cautious.

Overall, the study highlights the value of a facet-based and integrative approach to understanding emotional processing in psychopathy, with potential implications for assessment and intervention in forensic contexts.

## Figures and Tables

**Table 1:** Sociodemographic and juridical penal characterization (total, female, and male samples).

	Total Sample		Women		Men		*χ* ^2^	*p*	*V*
	N	%	N	%	N	%			
Marital status									
Single	140	54.5	55	48.7	85	59.0	4.502	0.212	0.132
Divorced/separated	45	17.5	19	16.8	26	18.1			
Married/civil union	62	24.1	33	29.2	29	20.1			
Widower	10	3.9	6	5.3	4	2.8			
Nationality									
Portuguese	232	90.3	94	83.2	138	95.8	11.533	<0.001	0.212
Other	25	9.7	19	16.8	6	4.2			
Educational level									
Illiterate	3	1.2	3	2.7	0	0	29.885	<0.001	0.341
4th grade	34	13.2	26	23.0	8	5.6			
6th grade	65	25.3	20	17.7	45	31.3			
9th grade	74	28.8	24	21.2	50	34.7			
12th grade	66	25.8	35	31.0	31	21.5			
Graduation	15	5.8	5	4.4	10	6.9			
Legal status									
Convicted	213	82.9	89	78.8	124	86.1	2.411	0.121	0.097
Remand	44	17.1	24	21.2	20	13.9			
Reoffense status									
First offender	160	62.3	84	74.3	76	52.8	12.523	<0.001	0.221
Repeat offender	97	37.7	29	25.7	68	47.2			
	Total sample		Women		Men				
	M	SD	M	SD	M	SD	*t*	*p*	*d*
Age	40.71	11.23	42.74	11.21	39.13	11.03	2.583	0.010	0.574
Sentence length	95.49	74.27	93.39	67.06	96.92	79.03	−0.341	0.733	0.225

**Table 2:** Comparison of psychopathy (total and factors scores) and emotional processing between sexes, controlling for age.

	Total Sample		Women		Men		*F*	*p*	η^2^_p_
	M	SD	M	SD	M	SD			
SRP-SF	63.52	18.79	57.10	16.56	68.56	18.96	30.850	<0.001	0.196
INT	14.45	5.34	12.81	5.06	15.74	5.21	18.557	<0.001	0.128
AFF	13.99	4.40	12.95	4.07	14.81	4.48	14.529	<0.001	0.103
LIF	17.00	6.03	15.27	5.43	18.35	6.15	31.473	<0.001	0.199
ANT	14.98	5.60	13.30	4.54	16.30	6.00	17.978	<0.001	0.124
EPS-25	4.53	1.91	4.86	2.01	3.73	1.60	2.518	0.083	0.020
SUP	5.21	2.35	5.58	2.63	4.91	2.05	2.604	0.076	0.020
UNP	5.09	2.44	5.55	2.66	4.72	2.18	3.843	0.023	0.030
CONT	3.37	2.46	3.36	2.43	3.38	2.43	1.789	0.169	0.014
AVO	4.96	2.03	5.21	2.22	4.76	1.84	3.779	0.024	0.029
IMP	4.00	2.37	4.40	2.52	3.67	2.20	4.338	0.014	0.034

Note. After Benjamini–Hochberg (B-H) correction effects with *p* ≤ 0.024 remained statistically significant. INT = Interpersonal; AFF = Affective; LIF = Lifestyle; ANT = Antisocial; SUP = Suppression; UNP = Unprocessed Emotion; CONT = Controllability; AVO = Avoidance; IMP = Impoverished Emotional Expression.

**Table 3:** Pearson Correlation Analysis Between Psychopathy and Emotional Processing for Total Sample, Men, and Women.

Total Sample	1	2	3	4	5	6	7	8	9	10
1. Psychopathy	1									
2. Interpersonal	0.856 ***	1								
3. Affective	0.817 ***	0.669 ***	1							
4. Lifestyle	0.889 ***	0.665 ***	0.654 ***	1						
5. Antisocial	0.844 ***	0.616 ***	0.544 ***	0.696 ***	1					
6. Emotional processing	0.156 *	0.147 *	0.223 ***	0.145 *	0.064	1				
7. Suppression	0.037	0.039	0.059	0.033	0.028	0.769 ***	1			
8. Unprocessed emotion	0.046	0.039	0.092	0.065	−0.008	0.869 ***	0.633 ***	1		
9. Controllability	0.394 ***	0.327 **	0.412 ***	0.393 ***	0.256 ***	0.789 ***	0.415 ***	0.588 ***	1	
10. Avoidance	0.027	0.059	0.096	0.004	−0.037	0.800 ***	0.521 ***	0.640 ***	0.535 ***	1
11. Impoverished expression	0.113	0.122	0.237 ***	0.072	0.006	0.876 ***	0.586 ***	0.691 ***	0.670 ***	0.641 ***
Men	1	2	3	4	5	6	7	8	9	10
1. Psychopathy	1									
2. Interpersonal	0.891 ***	1								
3. Affective	0.792 ***	0.694 ***	1							
4. Lifestyle	0.870 ***	0.674 ***	0.615 ***	1						
5. Antisocial	0.790 ***	0.629 ***	0.422 ***	0.613 ***	1					
6. Emotional processing	0.082	0.124	0.146	0.058	−0.019	1				
7. Suppression	−0.018	0.055	0.023	−0.080	−0.021	0.795 ***	1			
8. Unprocessed emotion	−0.038	−0.008	−0.055	0.007	−0.072	0.863 ***	0.636 ***	1		
9. Controllability	0.299 ***	0.282 **	0.376 ***	0.301 **	0.089	0.809 ***	0.443 ***	0.629 ***	1	
10. Avoidance	0.005	0.075	0.082	−0.044	−0.074	0.825 ***	0.579 ***	0.639 ***	0.591 ***	1
11. Impoverished expression	0.097	0.120	0.197 *	0.056	−0.006	0.900 ***	0.652 ***	0.694 ***	0.732 ***	0.695 ***
Women	1	2	3	4	5	6	7	8	9	10
1. Psychopathy	1									
2. Interpersonal	0.814 ***	1								
3. Affective	0.819 ***	0.620 ***	1							
4. Lifestyle	0.887 ***	0.620 ***	0.648 ***	1						
5. Antisocial	0.856 ***	0.568 ***	0.572 ***	0.709 ***	1					
6. Emotional processing	0.323 ***	0.262 **	0.366 ***	0.304 ***	0.203 *	1				
7. Suppression	0.177 *	0.109	0.157	0.210 *	0.141	0.727 ***	1			
8. Unprocessed emotion	0.223 **	0.183 *	0.298 ***	0.208 *	0.121	0.869 ***	0.611 ***	1		
9. Controllability	0.496 ***	0.384 ***	0.456 ***	0.484 ***	0.380 ***	0.791 ***	0.400 ***	0.569 ***	1	
10. Avoidance	0.116	0.112	0.159	0.100	0.040	0.763 ***	0.432 ***	0.629 ***	0.491 ***	1
11. Impoverished expression	0.227 **	0.220 **	0.348 ***	0.165	0.087	0.846 ***	0.492 ***	0.672 ***	0.630 ***	0.570 ***

Note. * *p* < 0.05; ** *p* < 0.01; *** *p* < 0.001.

**Table 4:** Hierarchical Regression Analyses Predicting Emotional Processing: Main Effects of Psychopathic Traits (total score and facets) and Moderating Effects of Sex.

	***β***	***t***	***p***	**95% CI**
Age	0.115	1.751	0.081	[−0.002; 0.042]
Nationality	0.022	0.362	0.718	[−0.082; 0.119]
Educational level	−0.171	−2.760	0.006	[−0.483; −0.081]
Sex	0.069	1.056	0.292	[−0.236; 0.781]
Reoffending status	−0.178	−2.741	0.007	[−1.175; −0.192]
Psychopathy	0.161	1.506	0.133	[−0.005; 0.038]
Sex × Psychopathy	0.115	1.147	0.253	[−0.011; 0.041]
	***β***	***t***	***p***	**95% CI**
Age	0.082	1.300	0.195	[−0.007; 0.035]
Nationality	0.024	0.398	0.691	[−0.081; 0.121]
Educational level	−0.169	−2.708	0.007	[−0.479; −0.076]
Sex	0.116	1.830	0.068	[−0.035; 0.949]
Reoffending status	−0.186	−2.857	0.005	[−1.206; −0.222]
Interpersonal	0.194	1.969	0.050	[0.000; 0.139]
Sex × Interpersonal	0.041	0.437	0.662	[−0.070; 0.110]
	***β***	***t***	***p***	**95% CI**
Age	0.101	1.621	0.106	[−0.004; 0.039]
Nationality	−0.004	−0.061	0.951	[−0.103; 0.096]
Educational level	−0.148	−2.421	0.016	[−0.440; −0.045]
Sex	0.101	1.607	0.109	[−0.090; 0.890]
Reoffending status	−0.176	−2.775	0.006	[−1.158; −0.197]
Affective	0.212	2.146	0.033	[0.008; 0.176]
Sex × Affective	0.085	0.878	0.381	[−0.060; 0.155]
	***β***	***t***	***p***	**95% CI**
Age	0.123	1.826	0.069	[−0.002; 0.044]
Nationality	0.037	0.611	0.542	[−0.070; 0.132]
Educational level	−0.179	−2.857	0.005	[−0.499; −0.092]
Sex	0.062	0.937	0.350	[−0.271; 0.762]
Reoffending status	−0.161	−2.510	0.013	[−1.104; −0.133]
Lifestyle	0.147	1.370	0.172	[−0.020; 0.113]
Sex × Lifestyle	0.123	1.240	0.216	[−0.030; 0.131]
	***β***	***t***	***p***	**95% CI**
Age	0.051	0.791	0.430	[−0.013; 0.031]
Nationality	0.020	0.327	0.744	[−0.086; 0.120]
Educational level	−0.152	−2.411	0.017	[−0.455; −0.046]
Sex	0.107	1.564	0.119	[−0.109; 0.952]
Reoffending status	−0.141	−2.143	0.033	[−1.043; −0.044]
Antisocial	−0.041	−0.352	0.725	[−0.093; 0.065]
Sex × Antisocial	0.047	1.366	0.173	[−0.029; 0.158]

Note. Only the results from the final model (Model 3), which included all variables and the interaction term, are presented in the table. Specifically, Model 1 included age, nationality, education, crime type, and sex as covariates; Model 2 added total psychopathy scores (or, in separate analyses, psychopathy facets); and Model 3 further included the interaction term between psychopathy and sex (psychopathy × sex).

## Data Availability

Due to the sensitive nature of the data, which includes personal and psychological information from incarcerated women, raw data are not publicly available to protect participant confidentiality. De-identified data are available from the corresponding author, DR, upon reasonable request, subject to ethical and legal approval.

## References

[B1-behavsci-16-01160] Anton M., Baskin–Sommers A., Vitale J., Curtin J., Newman J. (2018). Differential effects of psychopathy and antisocial personality disorder symptoms on cognitive and fear processing in female offenders. Cognitive, Affective, & Behavioral Neuroscience.

[B2-behavsci-16-01160] Bailey B. A. (2010). Partner violence during pregnancy: Prevalence, effects, screening, and management. International Journal of Women’s Health.

[B3-behavsci-16-01160] Baker R., Thomas S., Thomas P. W., Gower P., Santonastaso M., Whittlesea A. (2010). The emotional processing scale: Scale refinement and abridgement (EPS-25). Journal of Psychosomatic Research.

[B4-behavsci-16-01160] Beryl R., Chou S., Vollm B. (2014). A systematic review of psychopathy in women within secure settings. Personality and Individual Differences.

[B5-behavsci-16-01160] Blair R. J. (2005). Responding to the emotions of others: Dissociating forms of empathy through the study of typical and psychiatric populations. Consciousness and Cognition.

[B6-behavsci-16-01160] Blair R. J. (2007). The amygdala and ventromedial prefrontal cortex in morality and psychopathy. Trends in Cognitive Sciences.

[B7-behavsci-16-01160] Boduszek D., Dhingra K., Debowska A. (2016). The moderating role of psychopathic traits in the relationship between period of confinement and criminal social identity in a sample of juvenile prisoners. Journal of Criminal Justice.

[B8-behavsci-16-01160] Bonta J., Andrews D. A. (2023). The psychology of criminal conduct.

[B9-behavsci-16-01160] Brook M., Brieman C. L., Kosson D. S. (2013). Emotion processing in Psychopathy Checklist—Assessed psychopathy: A review of the literature. Clinical Psychology Review.

[B10-behavsci-16-01160] Cale E. M., Lilienfeld S. O. (2002). Sex differences in psychopathy and antisocial personality disorder. A review and integration. Clinical Psychology Review.

[B11-behavsci-16-01160] Campbell M. A., French S., Gendreau P. (2009). The prediction of violence in adult offenders: A meta-analytic comparison of instruments and methods of assessment. Criminal Justice and Behavior.

[B12-behavsci-16-01160] Cleckley H. (1941). The mask of sanity.

[B13-behavsci-16-01160] Cloitre M., Stolbach B. C., Herman J. L., van der Kolk B., Pynoos R., Wang J., Petkova E. (2009). A developmental approach to complex PTSD: Childhood and adult cumulative trauma as predictors of symptom complexity. Journal of Traumatic Stress.

[B14-behavsci-16-01160] Cohen J. (1988). Statistical power analysis for the behavioral sciences.

[B15-behavsci-16-01160] Cunha O., Sousa M., Almeida T. C., Guarda R., Cruz A. R. (2025). When good experiences matter: Positive childhood experiences as a moderator between adverse childhood experiences and psychopathic traits in community and justice-involved samples. Journal of Aggression, Maltreatment & Trauma.

[B16-behavsci-16-01160] De Brito S. A., Forth A. E., Baskin-Sommers A. R., Brazil I. A., Kimonis E. R., Pardini D., Frick P. J., Blair R. J. R., Viding E. (2021). Psychopathy. Nature Reviews Disease Primers.

[B17-behavsci-16-01160] de Vogel V., Lancel M. (2016). Gender differences in the assessment and manifestation of psychopathy: Results from a multicenter study in forensic psychiatric patients. International Journal of Forensic Mental Health.

[B18-behavsci-16-01160] de Vogel V., Nicholls T. L. (2016). Gender matters: An introduction to the special issues on women and girls. International Journal of Forensic Mental Health.

[B19-behavsci-16-01160] Dolan M., Fullam R. (2006). Face affect recognition deficits in personality-disordered offenders: Association with psychopathy. Psychological Medicine.

[B20-behavsci-16-01160] Edens J. F., Marcus D. K., Lilienfeld S. O., Poythress N. G. (2006). Psychopathic, not psychopath: Taxometric evidence for the dimensional structure of psychopathy. Journal of Abnormal Psychology.

[B21-behavsci-16-01160] Edwards B. G., Ermer E., Salovey P., Kiehl K. A. (2019). Emotional intelligence in incarcerated female offenders with psychopathic traits. Journal of Personality Disorders.

[B22-behavsci-16-01160] Falkenbach D. M., Reinhard E. E., Larson F. R. R. (2017). Theory based gender differences in psychopathy subtypes. Personality and Individual Differences.

[B23-behavsci-16-01160] Fine J. G., Semrud-Clikeman M., Zhu D. C. (2009). Gender differences in BOLD activation to face photographs and video vignettes. Behavioural Brain Research.

[B24-behavsci-16-01160] Ford J. D., Courtois C. A. (2014). Complex PTSD, affect dysregulation, and borderline personality disorder. Borderline Personality Disorder and Emotion Dysregulation.

[B25-behavsci-16-01160] Forouzan E., Cooke D. J. (2005). Figuring out la femme fatale: Conceptual and assessment issues concerning psychopathy in females. Behavioral Sciences & the Law.

[B26-behavsci-16-01160] Fowles D. C., Dindo L., Patrick C. J. (2006). A dual-deficit model of psychopathy. Handbook of psychopathy.

[B27-behavsci-16-01160] Fox B., DeLisi M. (2019). Psychopathic killers: A meta-analytic review of the psychopathy-homicide nexus. Aggression and Violent Behavior.

[B28-behavsci-16-01160] Fullam R., Dolan M. (2006). Emotional information processing in violent patients with schizophrenia: Association with psychopathy and symptomatology. Psychiatry Research.

[B29-behavsci-16-01160] Gaertner M., Sauter D., Baumgartl H., Rieg T., Buettner R. (2021). Multi-class emotion recognition within the valence-arousal-dominance space using EEG. 27th Americas Conference on Information Systems (AMCIS 2021).

[B30-behavsci-16-01160] Gonçalves L. C., Gonçalves R. A., Martins C., Dirkzwager A. J. E. (2014). Predicting infractions and health care utilization in prison: A meta-analysis. Criminal Justice and Behavior.

[B31-behavsci-16-01160] Gonçalves R. A. (2004). Adaptation to prison and psychopathy. Victims and offenders.

[B32-behavsci-16-01160] Gonzalez H. A., Muzaffar S., Yoo J., Elfadel I. M. (2020). BioCNN: A hardware inference engine for EEG-based emotion detection. IEEE Access.

[B33-behavsci-16-01160] Grann M. (2000). The PCL–R and gender. European Journal of Psychological Assessment.

[B34-behavsci-16-01160] Grieve R., Mahar D. (2010). The emotional manipulation–psychopathy nexus: Relationships with emotional intelligence, alexithymia and ethical position. Personality and Individual Differences.

[B35-behavsci-16-01160] Gross J. J., Jazaieri H. (2014). Emotion, Emotion Regulation, and Psychopathology: An Affective Science Perspective. Clinical Psychological Science.

[B36-behavsci-16-01160] Gross J. J., John O. P. (2003). Individual differences in two emotion regulation processes: Implications for affect, relationships, and well-being. Journal of Personality and Social Psychology.

[B37-behavsci-16-01160] Guay J. P., Knight R. A., Ruscio J., Hare R. D. (2018). A taxometric investigation of psychopathy in women. Psychiatry Research.

[B38-behavsci-16-01160] Guy L. S., Edens J. F., Anthony C., Douglas K. S. (2005). Does psychopathy predict institutional misconduct among adults? A meta-analytic investigation. Journal of Consulting and Clinical Psychology.

[B39-behavsci-16-01160] Hamilton R. B., Newman J. P., Patrick C. J. (2018). The response modulation hypothesis: Formulation, development, and implications for psychopathy. Handbook of psychopathy.

[B40-behavsci-16-01160] Hare R. D. (1978). Electrodermal and cardiovascular correlates of psychopathy. Psychopathic behavior: Approaches to research.

[B41-behavsci-16-01160] Hare R. D. (1980). A research scale for the assessment of psychopathy in criminal populations. Personality and Individual Differences.

[B42-behavsci-16-01160] Hare R. D. (2003). Manual for the revised psychopathy checklist.

[B43-behavsci-16-01160] Hare R. D., Neumann C. S. (2008). Psychopathy as a clinical and empirical construct. Annual Review of Clinical Psychology.

[B44-behavsci-16-01160] Hicks B. M., Markon K. E., Patrick C. J., Krueger R. F., Newman J. P. (2004). Identifying psychopathy subtypes on the basis of personality structure. Psychological Assessment.

[B45-behavsci-16-01160] Hoppenbrouwers S. S., Bulten B. H., Brazil I. A. (2016). Parsing fear: A reassessment of the evidence for fear deficits in psychopathy. Psychological Bulletin.

[B46-behavsci-16-01160] Hurezan L., Miu A. C., Costea A., Visu-Petra L. (2026). Dark personality, empathy, and prosocial behavior in convicted offenders. Scientific Reports.

[B47-behavsci-16-01160] Iria C., Barbosa F. (2009). Perception of facial expressions of fear: Comparative research with criminal and non-criminal psychopaths. The Journal of Forensic Psychiatry & Psychology.

[B48-behavsci-16-01160] Justus A. N., Finn P. R. (2007). Startle modulation in non-incarcerated men and women with psychopathic traits. Personality and Individual Differences.

[B49-behavsci-16-01160] Karpman B. (1948). The myth of the psychopathic personality. American Journal of Psychiatry.

[B50-behavsci-16-01160] Kret M. E., De Gelder B. (2012). A review on sex differences in processing emotional signals. Neuropsychologia.

[B51-behavsci-16-01160] Kret M. E., Ploeger A. (2015). Emotion processing deficits: A liability spectrum providing insight into comorbidity of mental disorders. Neuroscience & Biobehavioral Reviews.

[B52-behavsci-16-01160] Lauriola M., Donati M. A., Trentini C., Tomai M., Pontone S., Baker R. (2021). The structure of the emotional processing scale (EPS-25). European Journal of Psychological Assessment.

[B53-behavsci-16-01160] Lehmann A., Ittel E. (2012). Aggressive behavior and measurement of psychopathy in female inmates of German prisons—A preliminary study. International Journal of Law and Psychiatry.

[B54-behavsci-16-01160] Leistico A. M., Salekin R. T., DeCoster J., Rogers R. (2008). A large-scale meta-analysis relating the hare measures of psychopathy to antisocial conduct. Law and Human Behavior.

[B55-behavsci-16-01160] Lykken D. T. (1995). The antisocial personalities.

[B56-behavsci-16-01160] Łuczak E., Tanaś Ł. (2021). Psychopathic traits and empathy: Moral decision making and complex affect expression recognition. Psychiatria Polska.

[B57-behavsci-16-01160] Moffett S., Javdani S., Miglin R., Sadeh N. (2020). Examining latent profiles of psychopathy in a mixed-gender sample of juvenile detainees. Personality Disorders: Theory, Research, and Treatment.

[B58-behavsci-16-01160] Neumann C. S., Hare R. D., Newman J. P. (2007). The superordinate nature of the psychopathy checklist-revised. Journal of Personality Disorders.

[B59-behavsci-16-01160] Neumann C. S., Schmitt D. S., Carter R., Embley I., Hare R. D. (2012). Psychopathic traits in females and males across the globe. Behavioral Sciences & the Law.

[B60-behavsci-16-01160] Newman J. P., Curtin J. J., Bertsch J. D., Baskin-Sommers A. R. (2010). Attention moderates the fearlessness of psychopathic offenders. Biological Psychiatry.

[B61-behavsci-16-01160] Newman J. P., Kosson D. S. (1986). Passive avoidance learning in psychopathic and nonpsychopathic offenders. Journal of Abnormal Psychology.

[B62-behavsci-16-01160] Oskarsson S., Patrick C. J., Siponen R., Bertoldi B. M., Evans B., Tuvblad C. (2021). The startle reflex as an indicator of psychopathic personality from childhood to adulthood: A systematic review. Acta Psychologica.

[B63-behavsci-16-01160] Patrick C. J. (1994). Emotion and psychopathy: Startling new insights. Psychophysiology.

[B64-behavsci-16-01160] Patrick C. J., Rottenberg J., Johnson S. L. (2007). Affective processes in psychopathy. Emotion and psychopathology: Bridging affective and clinical science.

[B65-behavsci-16-01160] Patrick C. J. (2018). Psychopathy as masked pathology. Handbook of psychopathy.

[B66-behavsci-16-01160] Patrick C. J., Fowles D. C., Krueger R. F. (2009). Triarchic conceptualization of psychopathy: Developmental origins of disinhibition, boldness, and meanness. Development and Psychopathology.

[B67-behavsci-16-01160] Pinheiro M., Cunha O., Gonçalves R. A. (2020). Emotions, affections and psychopathy among female prisoners. International Journal of Offender Therapy and Comparative Criminology.

[B68-behavsci-16-01160] Pinheiro M., Gonçalves R. A., Cunha O. (2021). Criminal lifestyle, psychopathy, and prison adjustment among female inmates. Journal of Criminal Justice.

[B69-behavsci-16-01160] Pinheiro M., Gonçalves R. A., Cunha O. (2022). Psychopathy and reoffending among incarcerated women. Women and Criminal Justice.

[B70-behavsci-16-01160] Pinheiro M., Gonçalves R. A., Cunha O. (2024). Emotional processing and psychopathy among women: A systematic review. Deviant Behavior.

[B71-behavsci-16-01160] Pinheiro M., Gonçalves R. A., Cunha O. (2025). Emotional processing of pictures and psychopathic traits in women who committed crimes. Women & Criminal Justice.

[B72-behavsci-16-01160] Rada C., Forțu A. C. (2026). Personality dimensions Involved in the adaptation to the prison environment: Evidence from Romanian violent offenders. Social Sciences.

[B73-behavsci-16-01160] Schienle A., Schafer A., Stark R., Walter B., Vaitl D. (2005). Gender differences in the processing of disgust- and fear-inducing pictures: An fMRI study. Neuroreport.

[B74-behavsci-16-01160] Seara-Cardoso A., Queirós A., Fernandes E., Coutinho J., Neumann C. (2019). Psychometric properties and construct validity of the short version of the self-report psychopathy scale in a southern European sample. Journal of Personality Assessment.

[B75-behavsci-16-01160] Sellbom M., Drislane L. E. (2021). The classification of psychopathy. Aggression and Violent Behavior.

[B76-behavsci-16-01160] Serin R. C., Chadwick N., Lloyd C. D. (2016). Dynamic risk and protective factors. Psychology, Crime & Law.

[B77-behavsci-16-01160] Singh J. P., Grann M., Fazel S. (2011). A comparative study of violence risk assessment tools: A systematic review and metaregression analysis of 68 studies involving 25,980 participants. Clinical Psychology Review.

[B78-behavsci-16-01160] Skeem J. L., Cooke D. J. (2010). Is criminal behavior a central component of psychopathy? Conceptual directions for resolving the debate. Psychological Assessment.

[B79-behavsci-16-01160] Skeem J. L., Johansson P., Andershed H., Kerr M., Louden J. E. (2007). Two subtypes of psychopathic violent offenders that parallel primary and secondary variants. Journal of Abnormal Psychology.

[B80-behavsci-16-01160] Skilling T. A., Quinsey V. L., Craig W. M. (2001). Evidence of a taxon underlying serious antisocial behavior in boys. Criminal Justice and Behavior.

[B81-behavsci-16-01160] Snowden R. J., Frongillo Juric A., Leach R., McKinnon A., Gray N. S. (2022). Automatic processing of emotional images and psychopathic personality traits. Cognition and Emotion.

[B82-behavsci-16-01160] Sousa M., Cunha O., Gonçalves T., Gonçalves R. A., de Castro Rodrigues A. (2026). From abuse to offense: The legacy of childhood sexual abuse on the psychological functioning of men who perpetrated child sexual abuse. Victims & Offenders.

[B83-behavsci-16-01160] Sousa M., Gouveia C., Freitas B., Caridade S., Cunha O. (2025). The effectiveness of psychological intervention for women who committed child sexual abuse: An empty systematic review. Trauma, Violence & Abuse.

[B84-behavsci-16-01160] Thomson N. D., Kiehl K. A., Bjork J. M. (2019). Violence and aggression in young women: The importance of psychopathy and neurobiological function. Physiology & Behavior.

[B85-behavsci-16-01160] Thomson N. D., Towl G. J., Centifanti L. C. M. (2016). The habitual female offender inside: How psychopathic traits predict chronic prison violence. Law and Human Behavior.

[B86-behavsci-16-01160] van Wingen G. A., Geuze E., Vermetten E., Fernandez G. (2011). Perceived threat predicts the neural sequelae of combat stress. Molecular Psychiatry.

[B87-behavsci-16-01160] Verona E., Bresin K., Patrick C. J. (2013). Revisiting psychopathy in women: Cleckley/Hare conceptions and affective response. Journal of Abnormal Psychology.

[B88-behavsci-16-01160] Verona E., Vitale J. (2006). Psychopathy in women: Assessment, manifestations, and etiology. Handbook of psychopathy.

[B89-behavsci-16-01160] Verona E., Vitale J., Patrick C. J. (2018). Psychopathy in women: Assessment, manifestations, and etiology. Handbook of psychopathy.

[B90-behavsci-16-01160] Vitale E. J., Smith S., Brinkley A., Newman P. (2002). The reliability and validity of the Psychopathy Checklist–Revised in a sample of female offenders. Criminal Justice and Behavior.

[B91-behavsci-16-01160] Walters G. D., Polaschek D. L. L., Day A., Hollin C. R. (2019). Criminal thinking: Theory and practice. The Wiley international handbook of correctional psychology.

[B92-behavsci-16-01160] Whittle S., Yucel M., Yap M. B. H., Allen N. B. (2011). Sex differences in the neural correlates of emotion: Evidence from neuroimaging. Biological Psychology.

[B93-behavsci-16-01160] Willis G. M., Ward T., Craig L. A., Dixon L., Gannon T. A. (2024). Evidence for the good lives model in supporting rehabilitation and desistance from offending. The Wiley handbook of what works in correctional rehabilitation: An evidence-based approach to theory, assessment and treatment.

[B94-behavsci-16-01160] Wynn R., Høiseth M. H., Pettersen G. (2012). Psychopathy in women: Theoretical and clinical perspectives. International Journal of Women’s Health.

